# Sustained Complete Response to Brigatinib in a Young Patient With ALK‐Positive NSCLC Harboring I1171N Mutation Post‐Alectinib Resistance

**DOI:** 10.1155/crom/7372418

**Published:** 2026-02-19

**Authors:** Akhil Kapoor, Ajay Kumar Singh, Mahesh Chaudhary, Anuj Gupta, Bipinesh Sansar, Bal Krishna Mishra, Shashikant Patne, Arvind Suresh

**Affiliations:** ^1^ Department of Medical Oncology, Homi Bhabha Cancer Hospital and Mahamana Pandit Madan Mohan Malaviya Cancer Centre, Tata Memorial Centre, Homi Bhabha National Institute, Varanasi, India, hbni.ac.in; ^2^ Department of Medical Oncology, Paras Hospital, Kanpur, India; ^3^ Department of Pathology, Homi Bhabha Cancer Hospital and Mahamana Pandit Madan Mohan Malaviya Cancer Centre, Tata Memorial Centre, Homi Bhabha National Institute, Varanasi, India, hbni.ac.in; ^4^ Department of Nuclear Medicine & Molecular Imaging, Homi Bhabha Cancer Hospital and Mahamana Pandit Madan Mohan Malaviya Cancer Centre, Tata Memorial Centre, Homi Bhabha National Institute, Varanasi, India, hbni.ac.in

## Abstract

ALK‐rearranged non–small cell lung cancer (NSCLC) represents a paradigm of precision oncology, but acquired resistance to second‐generation ALK tyrosine kinase inhibitors (TKIs) such as alectinib remains inevitable. We report a young, nonsmoker male with ALK‐positive metastatic NSCLC who achieved a sustained complete response (CR) to brigatinib following alectinib resistance mediated by the ALK I1171N mutation. After 22 months of response to alectinib, disease progression prompted repeat molecular profiling, which identified the I1171N alteration. Brigatinib was initiated, and a complete radiologic response was documented within 3 months and has been sustained for over 12 months, including durable intracranial disease control. Sustained CR in I1171N‐mediated alectinib resistance is rare and highlights the critical role of repeat molecular testing to guide ALK TKI sequencing.

## 1. Introduction

Non–small cell lung cancer (NSCLC) remains the leading cause of cancer‐related mortality globally [1]. ALK‐rearranged NSCLC represents a distinct molecular subset typically affecting younger, nonsmoking individuals with adenocarcinoma histology [2]. The introduction of ALK TKIs has transformed outcomes, with progressively potent agents available across treatment lines.

Alectinib remains widely used as first‐line therapy; however, acquired resistance inevitably emerges. While lorlatinib—now preferred in contemporary guidelines—offers broad coverage against diverse ALK resistance mutations, its neurocognitive and metabolic toxicities may limit real‐world use [3–5]. Consequently, many patients initially treated with alectinib still require molecularly informed sequencing upon progression.

The ALK I1171N mutation is a clinically significant secondary resistance alteration, arising in the *α*C‐helix of the kinase domain. It confers resistance to alectinib but retains sensitivity to brigatinib and lorlatinib. Identifying this mutation at progression is therefore essential to optimize treatment selection.

## 2. Case Presentation

A 26‐year‐old nonsmoker male presented in late 2021 with cough and chest discomfort. Imaging demonstrated a left lung mass, mediastinal lymphadenopathy, and a single subcentimeter asymptomatic cerebral metastasis. Given the lesion size and risk of radionecrosis, upfront radiotherapy was deferred.

## 3. Baseline Molecular Testing

A plasma‐based liquid biopsy assessing *EGFR*, *ALK*, *ROS1*, and other alterations was negative. The patient received four cycles of ABCP (atezolizumab, bevacizumab, carboplatin, and paclitaxel), but follow‐up imaging showed progressive pulmonary disease.

## 4. Tissue NGS Assessment

A tissue rebiopsy followed by next‐generation sequencing (Illumina platform, mean target coverage > 350×; hybrid capture panel covering major oncogenic drivers) revealed an EML4–ALK fusion (Variant 3). The negative liquid biopsy was attributed to low tumor DNA shedding, a recognized limitation of plasma assays.

## 5. First‐Line Targeted Therapy

**Figure 1:**
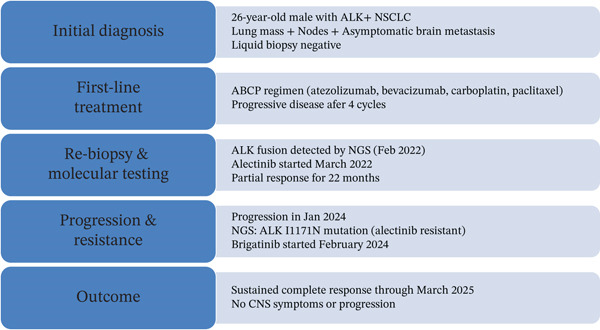
Figure summarizing the case timeline, molecular evolution, and treatment transitions.

**Figure 2:**
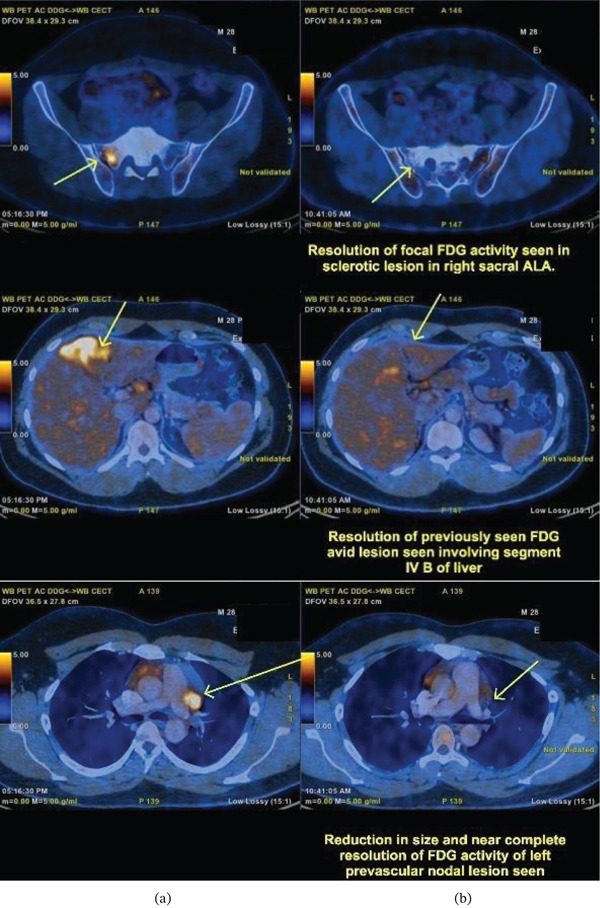
Response of PET‐CT scan of the patient showing comparison of (a) January 2024 scan images with (b) May 2024 scans. To note, brigatinib was started in February 2024 after the patient had progressive disease in the liver, nodes, and bones on alectinib.

**Figure 3:**
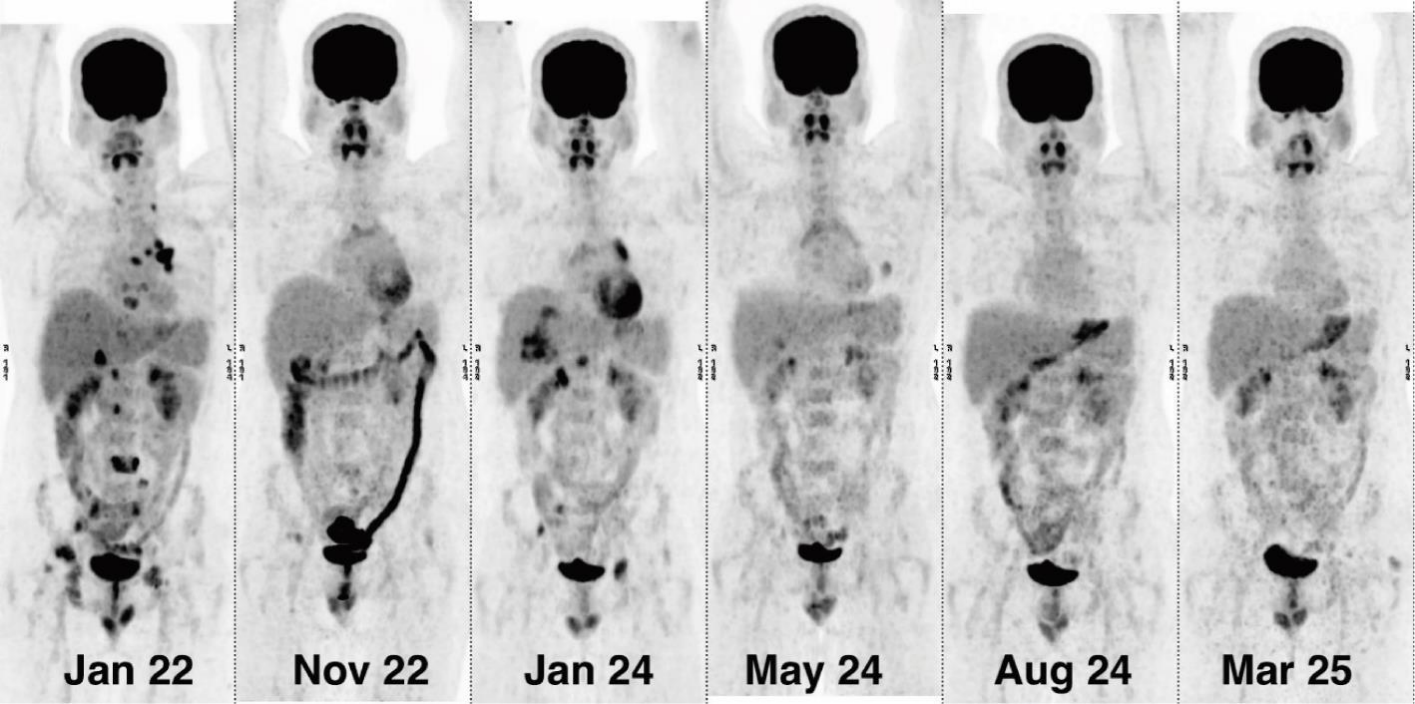
Serial scout images of FDG PET‐CT showing progressive liver, nodes, and bony lesions on January 24, which responded completely in the scan of May 24 after starting brigatinib, and the response was maintained in scans of August 24 and March 25.

Alectinib was initiated in March 2022. Figure 1 summarizes the case timeline, molecular evolution, and treatment transitions. A partial radiologic response occurred within 3 months, with a 42% reduction in target lesion size (Figure 2). Disease control was maintained for 22 months, as evidenced by serial scans (Figure 3).

## 6. Progression and Repeat Molecular Testing

In January 2024, imaging revealed progressive liver, nodal, and osseous lesions, while the brain remained stable. Repeat NGS (same platform and coverage) demonstrated the persistence of ALK fusion along with a newly acquired ALK I1171N mutation (VAF 24%). Brain MRI confirmed no intracranial progression. SRS/WBRT was not indicated.

## 7. Brigatinib Therapy and Treatment Response

Brigatinib was initiated in February 2024 (90 mg lead‐in ×7 days → 180 mg daily). Treatment was well tolerated, with only Grade 1 CPK elevation.

Follow‐up PET‐CT at 12 weeks showed a complete metabolic response with an approximately 90% reduction in overall tumor size, including near‐complete resolution of hepatic, nodal, and osseous lesions. Serial imaging in August 2024 and March 2025 confirmed a sustained complete response, with persistent metabolic inactivity and stable intracranial disease.

## 8. Discussion

ALK‐rearranged NSCLC exemplifies the success of precision oncology, with multiple generations of ALK TKIs offering sequential lines of targeted therapy. However, despite impressive responses to first‐line alectinib, most patients eventually develop acquired resistance. Resistance to alectinib is heterogeneous, arising from both ALK‐dependent and ALK‐independent mechanisms. Secondary ALK kinase domain mutations account for 30%–40% of cases and include alterations such as I1171N/T/S, V1180L, and G1202R [6]. The I1171N substitution in the *α*C‐helix alters the kinase conformation, reducing alectinib binding affinity. Importantly, preclinical and clinical data show preserved sensitivity to brigatinib and lorlatinib.

Brigatinib′s distinct structural and biochemical properties enable it to overcome resistance conferred by mutations such as I1171N, C1156Y, and L1196M. Additionally, brigatinib demonstrates robust central nervous system (CNS) penetration, a frequent site of disease progression in ALK‐positive NSCLC, making it a particularly effective option following alectinib failure [5, 7, 8]. The third‐generation ALK inhibitor, lorlatinib, offers the broadest coverage of ALK resistance mutations to date, including activity against the notoriously refractory G1202R mutation and several compound mutations [9]. However, its use may be constrained in certain patients by CNS‐related adverse effects, including cognitive and mood alterations, as well as dyslipidemia, which can limit its tolerability in real‐world settings. These considerations further elevate the need for precise molecular characterization at progression.

This case is noteworthy due to the patient′s young age and excellent long‐term responsiveness to sequential ALK‐targeted therapies. High‐quality tissue NGS confirmed I1171N‐mediated alectinib resistance, enabling a precise, mutation‐guided therapeutic shift. Following the initiation of brigatinib, the patient achieved a complete radiologic response within 3 months, which has been sustained for over 12 months, including continued intracranial disease stability. Durable complete responses in I1171N‐mediated alectinib resistance are rare, highlighting the unique clinical relevance of this case. However, it should be noted that this report lacks serial circulating tumor DNA (ctDNA) monitoring, prohibiting dynamic assessment of molecular evolution. Additionally, only single‐site tissue biopsies were analyzed, potentially underestimating tumor heterogeneity.

Table 1 summarizes the spectrum of resistance mutations in the ALK kinase domain and their differential sensitivity profiles. While lorlatinib retains efficacy against many single and compound mutations, recent data highlight that certain compound mutations involving I1171N or G1202R + L1196M may exhibit reduced sensitivity even to lorlatinib [10]. Intriguingly, earlier‐generation agents like brigatinib may retain activity in select lorlatinib‐resistant scenarios, particularly when compound mutations include I1171N. Table 2 details the mutation‐specific activity of brigatinib and lorlatinib, underscoring the complementary roles these agents play in sequential ALK inhibition.

**Table 1:** Activity of ALK tyrosine kinase inhibitors (TKIs) against known resistance mutations in the ALK kinase domain.

Mutation	Crizotinib	Ceritinib	Ensartinib	Alectinib	Brigatinib	Lorlatinib
I1171N/T/S	×	±	±	×	✓	✓
C1156Y	×	±	±	×	✓	✓
L1196M	×	✓	✓	×	✓	✓
V1180L	×	×	±	×	±	✓
G1202R	×	×	×	×	±	✓
F1174C/V	×	✓	✓	✓	✓	✓
G1269A	✓	✓	✓	✓	✓	✓
S1206Y	×	±	±	×	✓	✓
D1203N	×	±	±	±	✓	✓
Compound mutations	×	±	±	±	✓	±

**Table 2:** Activity of ALK inhibitors against selected compound mutations based on functional studies.

Compound mutation	Crizotinib	Alectinib	Ceritinib	Brigatinib	Lorlatinib
G1202R + L1198F	✓	×	×	×	×
I1171N + L1198F	✓	×	×	✓	×
I1171N + L1256F	×	✓	×	✓	×
I1171N + L1196M	×	×	✓	✓	×
I1171N + G1269A	×	×	✓	✓	×

Tailoring the sequence of ALK TKIs based on the molecular landscape at resistance is not only clinically rational but is increasingly supported by guideline recommendations and expert consensus. In our previously reported experience using a molecular tumor board approach, incorporation of repeat molecular testing—including tissue‐ or plasma‐based NGS—allowed for informed treatment selection and improved patient outcomes [9, 11].

Therefore, performing a repeat biopsy or ctDNA analysis at the time of disease progression is not merely a research endeavor—it is a clinical necessity. Without this, empiric sequencing of ALK TKIs may result in suboptimal outcomes, premature exposure to later‐line agents, and increased toxicity. Personalized therapy based on molecular resistance mechanisms represents the next frontier in maximizing outcomes in ALK‐positive NSCLC, and biopsy at progression is the gateway to that paradigm and may significantly improve outcomes when molecularly matched therapies are selected.

## Author Contributions

A.K. conceptualized the manuscript, drafted the manuscript, and provided the oncologic care. A.K.S. obtained the necessary images, provided the oncologic care, and contributed to editing the manuscript. M.C., A.G., B.S., B.K.M., S.P., and A.S. contributed to editing. B.K.M., A.G., and B.S. contributed to providing clinical care to the patient.

## Funding

No funding was received for this manuscript.

## Disclosure

All the authors approved the final manuscript. The manuscript does not have any identifiers of the patient. No other sources were utilized for the writing of this manuscript.

## Ethics Statement

Ethical approval was waived off by the institute since this is a case report of a patient treated as per the multidisciplinary tumor board.

## Consent

The patient provided written informed consent for the publication of this case report.

## Conflicts of Interest

The authors declare no conflicts of interest.

## General Statement

*Key Clinical Message.* This case emphasizes the utility of rebiopsy and molecular testing at progression to guide next‐line ALK TKI sequencing, resulting in a sustained and clinically meaningful response.

## Data Availability

Data sharing is not applicable to this article as no datasets were generated or analyzed for this study.
